# FDG uptake in cancer: a continuing debate

**DOI:** 10.7150/thno.40599

**Published:** 2020-02-06

**Authors:** Silvia Peppicelli, Elena Andreucci, Jessica Ruzzolini, Francesca Bianchini, Lido Calorini

**Affiliations:** 1 Department of Experimental and Clinical Biomedical Sciences “Mario Serio”, Section of Experimental Pathology and Oncology; 2 Interdepartmental Center for Molecular Preclinical Imaging Experimentation CISPIM, University of Florence; 3 Center of Excellence for Research, Transfer and High Education DenoTHE University of Florence, Florence, Italy

2-[^18^F]-fluoro-2-deoxy-D-glucose positron emission tomography/ computed tomography (FDG PET/CT) is routinely used in clinical oncology for the diagnosis of many different types of cancers, taking advantage of the high glucose avidity of neoplastic cells 1. It is indeed notable that, cancer cells display an “addiction” to “aerobic” glycolysis, the so-called “Warburg effect”. However, plasticity in the metabolic reprogramming of cancer cells is far more complex, as genetic and phenotypic changes lead to metabolic heterogeneity of tumor cell populations. Factors in the microenvironment, such as the level of nutrients, oxygen, pH, and the presence of host stromal cells may influence cancer cell metabolism. As a consequence, a low FDG uptake by cancer cells may be related to a low glycolytic rate or to the use of alternative substrates. However, in certain tumors, the diagnostic accuracy of FDG-PET may be affected either by the size of lesion (if below the spatial resolution of PET/CT), or by the physiological urinary excretion rate of the tracer, or by the relatively high metabolic activity of surrounding tissue. Indeed, in the case of bladder, prostate, kidney, breast and endocrine gland cancers, FDG/PET analysis is not recommended for diagnosis 2.

In order to improve the accuracy of FDG PET/CT imaging analysis, it is essential to seek further clarification in particular about the way in which cancer cells adapt to different metabolic profiles. In recent years, many authors have examined cancer metabolism and its reprogramming when cells are exposed to the influence of critical features of the tumor microenvironment, such as hypoxia and acidosis. Proliferating cancer cells use Warburg's aerobic glycolysis to move to “anaerobic” glycolysis when challenged by hypoxia. Both of these metabolic conditions lead to glucose exhaustion and low extracellular pH (pHe), due to lactic acid secretion associated with a reduction in the draining activity of the lymphatic system. Acidosis in the tumor microenvironment, in turn, reprograms cancer cell metabolism towards a more oxidative phenotype. Thus, the microenvironment may lead to different FDG avidity of cancer cells.

It has been reported quite recently that lactic acidosis inhibits breast cancer cell FDG uptake (e.g. in the human MDA-MB-231 triple-negative breast cancer cell line) 3. Single cell suspensions were obtained from core and peripheral regions of subcutaneous tumors removed from mice. Next, FDG uptake was determined in cells of the two regions by radioluminescence (RLM) imaging. FDG uptake was significantly higher in cells isolated from the periphery of the tumors than in those from the core. Accordingly, there was evidence of mitochondrial redox differences between the core and the periphery of tumors in human breast cancer mouse xenografts 4. Therefore, the microenvironment conditions which are considered capable of driving a change of FDG uptake are proliferation, hypoxia and acidosis. To corroborate RLM results further, the authors used gamma radiation counting, observing that the reduction of FDG uptake by cells from the core was promoted by either extracellular acidosis (both lactic and hydrochloric acidosis) or by sodium lactate, (although only at a high dosage). Several *in vitro* reports have shown that a transient (24 hour) 5 or a chronic (8-10 week) 6 exposure to an acidic medium (pH 6.7-6.8) causes a shift of cancer cell metabolism from glycolytic to respiratory. Accordingly, this shift is associated with a decrease of glucose transporters, thus accounting for the reduction of FDG uptake.

A further question is the case of oncogene-driven tumors. The constitutive activation of oncogene-dependent pathways might reinforce glycolytic metabolism and downregulate respiratory metabolism in cancer cells, via the reduction of both pyruvate kinase M2 (PKM2)-dependent conversion of phosphoenolpyruvate to pyruvate, and pyruvate flux to mitochondria 7. This might explain the reverse association, observed *in vivo*, between FDG uptake and extracellular pH (pHe) in human epidermal growth factor receptor-2-positive (HER2^+^) breast cancer grafts. In this case, HER2 regulates key effectors of cellular metabolism such as the serine/threonine kinase Akt, increasing glucose metabolism and explaining the acidic pHe. As a consequence, only the inhibition of oncogene expression could lead to a significant shift from glycolysis to oxidative phosphorylation.

Additional *in vivo* studies, dealing with the FGD uptake and pHe of cancer, have been carried out with conflicting results. In one study, the pHe of tumors, determined by chemical exchange saturation transfer (MRI-CEST) images, was found to be acidic (pH 6.7), and then FDG uptake was estimated by PET analysis. In order to validate CEST-pH mapping of tumors, animals were forced to drink bicarbonate-rich water to raise the pHe of tumors to almost neutral value (pH 7.0-7.1), demonstrating a significant increase of positive pixels in tumors from sodium bicarbonate-treated animals when compared to those from untreated animals. Upon this validation of the combination of PET/MRI-CEST images, the authors realized that tumors with higher FDG uptake were associated with a lower pHe, while tumors with lower FDG uptake displayed a less acidic microenvironment 8.

Although we could not exclude the possibility that some metabolic differences were related to the different tumors analyzed, it is very difficult to infer a clear conclusion from these data. After FGD phosphorylation, brought about by hexokinase or glucokinase, FDG-6P is not available for metabolic activity. This “metabolic trap” is fundamental for FDG/PET analysis. However, a low amount of intracellular FDG might be removed slowly from the cell by the action of 6-phosphatase on FDG-6P. An additional element of complexity is the possible decrease or absence of glucose-6-phosphatase (G6Pase) activity in tumor cells, which might play a role in the modulation of glucose fermentation, e.g. some tumor regions of a renal cancer with reduced G6Pase exhibit a high FDG uptake 9. The complex heterogeneity of cancer makes it difficult to predict a defined metabolic scenario.

Figure 1 describes the active pathways of various cancer cell subpopulations, considering changes of major nutrients and the distance from blood supply, which are the prominent drivers of metabolic adaptation. Because of this, cancer cells located in proximity of a blood vessel are usually proliferative, when both PI3K/Akt signaling and c-Myc promote glucose uptake and glycolysis, and the already mentioned “Warburg effect” is present. This metabolic profile produces enough ATP at a fast rate but also generates important intermediates necessary for cell division. Further, glycolysis yields pyruvate, which, together with glutamine, sustains the TCA cycle for additional lipid and amino acid biosynthesis. However, most of pyruvate (80%) is converted to lactate and then excreted by specific transporters; this is necessary to maintain glycolytic flux. Although vasculature might remove the majority of protons and/or lactate, limiting the pH reduction, the extracellular medium becomes acidic.

Moreover, in some tumor regions, despite the reduction in oxygen use, cancer cells move to HIF-1α-dependent anaerobic glycolysis when oxygen diffusion is limited and its percentage becomes less than 2%. In addition to this, the anaemia of cancer-bearing patients, occurring either as a paraneoplastic syndrome or as a common side effect of chemotherapy, contributes to the development of tissue hypoxia and to the activation of HIF-1α-dependent transcription factors. Major consequences of the switch to anaerobic glycolysis are a high and exclusive glucose uptake and the production of lactic acid, due to the HIF-1α promoting effect on glucose transporters and lactate dehydrogenase-A activity. The reduction of the proliferation level favors the resistance to apoptotic signals. Thus, both cancer cell sub-populations may acquire FDG but the anaerobic cells more so than the aerobic glycolytic cells. It should be noted that the tumor microenvironment contains host stromal cells, the so-called cancer-associated fibroblasts (CAF) which were observed to perform aerobic glycolysis (the so-called “reverse Warburg effect”), acquiring glucose and releasing lactate in normoxia 10. Therefore, hypoxic CAF, carrying out an anaerobic glycolysis, concur to consume glucose and discard lactate in the extracellular environment. Interestingly, CAF contribute to extend the heterogeneity of cancer cells promoting the FDG uptake in tumor cells, as observed by micro-PET analysis 11. Moreover, CAF influence FDG uptake in a model of non-small cell lung cancer. When the tumor-bearing animals were fed, FDG accumulated in noncancerous stroma of the tumors, whereas, when mice were made to fast, the predominant FDG uptake was ascribed to hypoxic cancer cells 12.

Furthermore, it has been demonstrated that the acidic microenvironment affects HIF-1α, which is the key glycolytic driver of hypoxic cancer cells. Indeed, when cancer cells are exposed, under hypoxic conditions (1% oxygen), to a slightly basic microenvironment (7.7 pHe), they express a very high level of HIF-1α as expected. The level of HIF-1α expression is not reduced when cells are exposed to 7.3 pHe, whereas it is severely reduced when cells are exposed to 6.8 pHe. HIF-1α completely disappears in cancer cells exposed to 6.3 pHe, under hypoxic conditions 13. In addition, during the early phase of tumor growth, when oxygen tension is still high enough (approx. 2.5% oxygen), pHe is already 6.6. When tumors are larger than 500 mm^3^, oxygen level is around 1% and the pHe is approximately 6.4, suggesting that low pHe not only influences hypoxic cancer cells but also the “Warburg-addicted” cancer cells of oxygenated regions 14. These findings indicate that acidosis, more than hypoxia, may represent the crucial factor in regulating cell metabolism, acting like a control trigger capable of switching cancer cell metabolism from glucose to other substrates.

Thus, a different degree of lactate accumulation may adjust the level of glucose uptake, as: (i) the low amount of lactate generated by low-glycolytic tumor cells does not modify the low level of FDG uptake; (ii) a medium amount of lactate, released by intermediate-glycolytic tumor cells, partially affects FDG uptake; (iii) a high amount of lactate, produced by high-glycolytic tumor/stromal cells, finally leads to a reduced glycolysis in adjacent tumor cell subpopulations, determining a significant reduction of FDG uptake.

The low pHe removes the inhibitory activity of the HIF-1α-dependent pyruvate dehydrogenase kinase-1 on pyruvate dehydrogenase, allowing the restoration of an electron flux through the oxidative phosphorylation. Thus, acidic cells, in a 0.5% (at least) oxygen microenvironment, may become oxidative using alternative substrates, whose lactate acquires a notable significance 15, 16.

In addition, acidic cancer cells reprogram their bioenergetic preferences toward glutamine and fatty acids, extending the range of their possible alternative sources 17. It is possible that chronically-addicted acidic cancer cells of a tumor mass, despite their reprogramming towards a more oxidative phosphorylation, maintain, in addition to the consumption of other substrates, some residual glucose uptake to be used for anaplerosis, which is critical for cell proliferation. Thus, chronic acidic cells might turn out to be weakly FDG positive.

Although the acidic cancer cells subpopulations express a more resistant and aggressive phenotype, often associated with an epithelial-to-mesenchymal transition, they are only weakly proliferative 18. Thus, we have suggested that an acidic microenvironment can constitute a perfect niche for dormant tumor cells endowed with all the characteristics useful for resistance to programmed cell death. Moreover, the niche of acidic cancer cells may support the re-expansion of cancer cells in the neighboring host tissues, which may be in search of new area of growth 19. It is possible that the new wave of tumor growth may start from the acidic/invasive front of the tumor, recapitulating a novel cell heterogeneity, indicating that an acidic microenvironment might be also a niche for the survival and renewal of cancer stem cells 20.

Beyond the points we have suggested here, there is still much to learn concerning tumor microenvironment. The rapid reprogramming of the metabolic profile of cancer cells is also involved in the emergence of a cancer stem cell-like phenotype 19, 20 and/or the acquisition of drug resistance 21, 22. A critical non-invasive evaluation of the different aspects of tumor microenvironment might predict cancer aggressiveness and patient prognosis 23, 24. Thus, in order to enhance the reliability of the crucial diagnostic tool represented by the use of FDG in medical imaging, we are convinced that the development of novel alternative tracers capable of estimating additional parameters such as intratumoral acidity should be pursued 25.

## Figures and Tables

**Figure 1:**
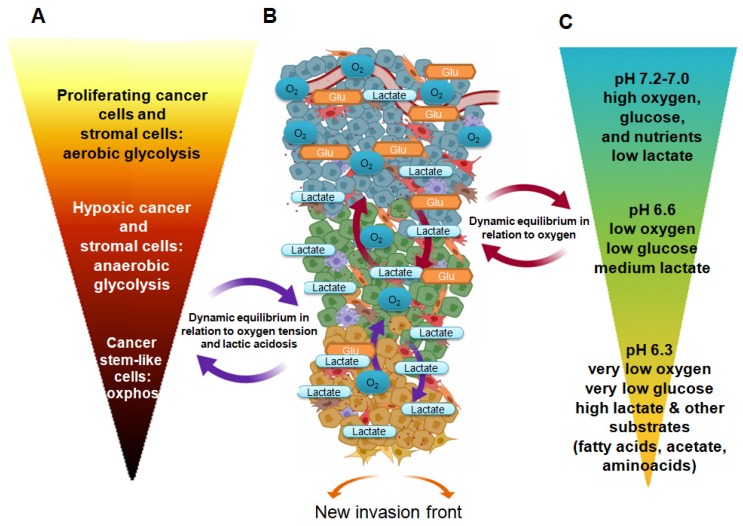
From proliferation to quiescence and aggressiveness: The figure describes the scenario of FDG uptake by the diverse metabolic cancer cell subpopulations inside a proliferative tumor. Changes in pHe and lactate due to local modification (e.g. high interstitial pressure, local ischemia) may drive a continuous metabolic reprogramming limiting FDG uptake. A, FDG uptake/ metabolism; B tumor microenvironment; C microenvironmental condition and nutrients.
